# Tyrosine Phosphorylation of Rac1: A Role in Regulation of Cell Spreading

**DOI:** 10.1371/journal.pone.0028587

**Published:** 2011-12-06

**Authors:** Fumin Chang, Christopher Lemmon, Daniel Lietha, Michael Eck, Lewis Romer

**Affiliations:** 1 Department of Anesthesiology and Critical Care Medicine, Johns Hopkins University School of Medicine, Baltimore, Maryland, United States of America; 2 Department of Cell Biology, Duke University Medical Center, Durham, North Carolina, United States of America; 3 Spanish National Cancer Research Centre (CNIO), Madrid, Spain; 4 Department of Biological Chemistry and Molecular Pharmacology, Harvard Medical School and Dana-Farber Cancer Institute, Boston, Massachusetts, United States of America; 5 Departments of Cell Biology, Biomedical Engineering, Pediatrics, and the Center for Cell Dynamics, Johns Hopkins University School of Medicine, Baltimore, Maryland, United States of America; Leiden University, The Netherlands

## Abstract

Rac1 influences a multiplicity of vital cellular- and tissue-level control functions, making it an important candidate for targeted therapeutics. The activity of the Rho family member Cdc42 has been shown to be modulated by tyrosine phosphorylation at position 64. We therefore investigated consequences of the point mutations Y64F and Y64D in Rac1. Both mutations altered cell spreading from baseline in the settings of wild type, constitutively active, or dominant negative Rac1 expression, and were accompanied by differences in Rac1 targeting to focal adhesions. Rac1-Y64F displayed increased GTP-binding, increased association with βPIX, and reduced binding with RhoGDI as compared with wild type Rac1. Rac1-Y64D had less binding to PAK than Rac1-WT or Rac1-64F. In vitro assays demonstrated that Y64 in Rac1 is a target for FAK and Src. Taken together, these data suggest a mechanism for the regulation of Rac1 activity by non-receptor tyrosine kinases, with consequences for membrane extension.

## Introduction

Rac1 is a member of the small guanosine triphosphatase Rho family of proteins which also includes Rho and Cdc42. Rac1 has been shown to play important roles in a wide variety of cellular processes, including cytoskeletal reorganization, cell migration, cell transformation, induction of DNA synthesis, superoxide production, and axonal guidance [1]–[8]. The classical understanding of the regulation of activity in Rho family members is based upon two conformations - the GTP-bound or “active” form, and the GDP-bound or “inactive” form [9]. Changes in Rac1 activation may be triggered by a variety of extracellular signals including matrix adhesion, growth factors, cytokines, and endocrine hormones, and by intracellular signals including cytosolic free calcium and lipid raft trafficking [10]–[13]. These signals are integrated via guanine nucleotide exchange factors (GEFs) which convert Rac1 from GDP bound to GTP bound form, and GTPase-activating proteins (GAPs), which convert GTP-bound to GDP-bound Rac1. Rho GDP-dissociation inhibitor (RhoGDI) also plays a regulatory role in Rac1 activity. RhoGDI is a cytosolic protein that associates with Rac1 and can prevent Rac1 from targeting to the cell membrane. RhoGDI therefore controls the access of Rac1 to regulatory GEFs and GAPs [14], [15].

Interestingly, the function of Rho family proteins may also be modulated via protein phosphorylation. Protein kinase A (PKA)-mediated phosphorylation of RhoA on Ser188 was observed both in vitro and in vivo in natural killer T lymphocytes [16]. This phosphorylation did not change RhoA GTPase activity or binding to GTP, but led to the exit of phosphorylated RhoA from the plasma membranes and an increased presence of the RhoA-RhoGDI complex in the cytosol. Increased cellular cAMP levels and PKA activity resulted in morphological changes consistent with RhoA inhibition. It was therefore suggested that PKA-mediated phosphorylation of RhoA inhibits Rho activity by promoting formation of a RhoA-RhoGDI complex. Similarly, PKA-mediated phosphorylation and a resultant increase in complex formation with RhoGDI was observed with both RhoA and Cdc42 in studies of rodent brain [17]. It is not clear whether Rac1 is a phosphorylation target for PKA, but Kwon et al. demonstrated phosphorylation of Rac1 on Ser-71 by Akt in human melanoma cells [18]. This Akt-mediated Rac1 phosphorylation resulted in an approximately 50% reduction in GTP binding by Rac1, but did not change GTPase activity. In the case of Cdc42, tyrosine phosphorylation at position 64 was observed following treatment with epidermal growth factor, and this was mediated by Src in COS-7 cells [19], [20]. Tyrosine-64 was identified as the major phosphorylation site in these experiments, but tyrosine phosphorylation on Y64 was not required for Cdc42 activation. Tyrosine phosphorylation on Y64 of Cdc42 also did not affect its binding with several target/effector proteins including PAK, ACK2, MRCK, WASP or IQGAP – but increased association with RhoGDI was noted. Since Cdc42-RhoGDI interactions are involved in Cdc42-induced cellular transformation, it was suggested that phosphorylation of Cdc42 led to alteration of its targeting via RhoGDI. The pattern that emerges from this earlier work is that protein phosphorylation may serve a specific role in signal modulation of Rho family GTPases by altering binding interactions with upstream regulators, with GTP, and with RhoGDI.

**Figure 1:**
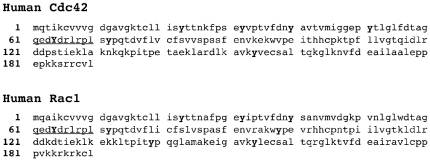
Sequence homology for Cdc42 and Rac1. Amino acid sequences are shown for human Cdc42 (top) and Rac1 (bottom). The sequence identity between the two proteins at aa 61–70 is underlined, Y64 is capitalized, and all tyrosine residues appear in bold type.

Tyrosine phosphorylation of Rac1 has not been explored to date, although we have demonstrated that tyrosine phosphorylation of βPIX is associated with increased binding to Rac1 in vitro, and augmentation of cell spreading [21]. Given that human Rac1 and Cdc42 share high homology and have the identical amino acid sequence at residues 61–70 (Figure 1), site-directed mutagenesis was used here to investigate the impact of Tyr-64 phosphorylation on cell spreading and the interaction of Rac1 with regulatory and effector proteins. Rac1-Y64F was used to obviate phosphorylation at this site, while Rac1-Y64D was employed to mimic the constitutively phosphorylated state. Strikingly, expression of the Rac1-Y64D mutant greatly inhibited cell spreading and decreased Rac1 binding to PAK. Expression of the Rac1-Y64F mutant facilitated cell spreading, while it increased Rac1 binding to GTP and to Rac1-associated GEFs, and decreased binding to RhoGDI. Specific interactions in vitro and in vivo indicated that Rac1 may be a substrate for the non-receptor tyrosine kinases FAK and Src.

## Methods

### Cell culture, DNA constructs, and transfection

Mouse embryo fibroblasts (MEFs), obtained from ATCC (Rockville, MD), and murine fibroblasts homozygous for deletion of the *Src*, *Yes and Fyn* genes (SYF) (a generous gift from Dr. Philippe Soriano, Mount Sinai School of Medicine of New York University) were maintained in Dulbecco's modified medium (DMEM) (Sigma, St Louis, MO) supplemented with 10% fetal bovine serum (Atlanta Biological, Atlanta, GA), 2 mM L-glutamine (Gibco-BRL, Grand Island, NY), penicillin G, streptomycin and amphotericin B (Gibco-BRL). HUVEC from VEC Technologies (Rensselaer, NY) were maintained in MCDB-131 complete medium (VEC). EGFP-tagged Rac1-WT (wild type), Rac1-61L and Rac1-17N were gifts from Dr. Klaus Hahn (University of North Carolina). GST-tagged Rac1-WT , Rac1-61L, and Rac1-17N were gifts from Dr. Ian Macara (University of Virginia). Wild-type and kinase dead Src constructs were gifts from Dr. Uyen Huynh-Do (University of Bern, Switzerland).

EGFP-tagged Rac1-Y64F and Rac1-Y64D mutants were constructed using the QuikChange Site-directed mutagenesis kit (Stratagene, La Jolla, CA) with EGFP-Rac1-WT as the template and the following primer sets: For Rac1-Y64D 5′-GGGACACAGCTGGACAAGAAGACGAT GACAGATTGCGTCCCC-3′ and 5′-GGGGACGCAATCTGTCATCGTCTTCTT GTCCAGCTGTGTCCC-3′; and for Rac1-Y64F 5′-GGGACACAGCTGGACAAGAAGACTTT GACAGATTGCGTCCCC-3′ and 5′-GGGGACGCAATCTGTCAAAGTCTTCTT GTCCAGCTGTGTCCC-3′ GST-tagged Rac1-Y64D and Rac1-Y64F were constructed with the same primer sets but with GST-Rac1-WT as the template.

Using EGFP-Rac1-17N as the template, the double mutants EGFP-Rac1-17N/64D and EGFP-Rac1-17N/64F were constructed with the same primer sets. EGFP-Rac1-61L/64D and EGFP-Rac1-61L/64F were constructed using EGFP-Rac1-61L as the template and the following primer sets: For 61L64D 5′-GGGACACAGCTGGACTAGAAGACGAT GACAGATTGCGTCCCC-3′ and 5′-GGGGACGCAATCTGTCATCGTCTTCTA GTCCAGCTGTGTCCC-3′; and for 61L64F 5′-GGGACACAGCTGGACTAGAAGACTTT GACAGATTGCGTCCCC-3′ and 5′-GGGGACGCAATCTGTCAAAGTCTTCTA GTCCAGCTGTGTCC-3′. GST-tagged double mutants were constructed with the same primer sets but with GST-Rac1-17N or GST-Rac1-61L as the templates. All new constructs were confirmed by both restriction enzyme digestion and DNA sequencing.

The avian FAK kinase domain (amino acids 411–686), and the Src kinase domain (amino acids 86–536, SH3-SH2-Kinase) [22]) were generated in baculovirus-transfected Hifive insect cells.

Transient transfections were accomplished using Lipofectamine Plus (Gibco-BRL) and standard product protocols. Briefly, cells were plated 24 hours before transfection. Cells were washed and then incubated for 3 hours in serum-free DMEM medium containing plasmid DNA mixed with Lipofectamine and Plus reagent. The medium was then replaced with DMEM containing 10% FBS and incubated for 48 hours before cells were prepared for immunofluorescence or immune replica analysis.

### Antibodies

Anti-Rac1 antibodies included mouse monoclonal clone 102 (BD Biosciences, San Diego CA) and rabbit polyclonal clone C-11 (Santa Cruz Biotechnology, Inc., Santa Cruz, CA). Other primary antibodies included anti-Src (rabbit polyclonal, Cell Signaling, Danvers, MA), anti-GFP (rabbit polyclonal, Abcam Inc., Cambridge MA), anti-GST (rabbit polyclonal, Molecular Probes, Eugene, OR), anti-Flag (rabbit polyclonal, Sigma, St. Louis, MO), anti-Myc (mouse monoclonal, Santa Cruz Biotechnology, Inc), anti-phosphotyrosine py20 (BD Biosciences), anti-cleaved caspase3 (Cell Signaling) and anti-vinculin (mouse monoclonal clone 7F9, a gift from Dr. Alexey Belkin, University of Maryland, Baltimore, MD). Horseradish peroxidase-conjugated anti-mouse and anti-rabbit secondary antibodies were obtained from ICN Biochemicals Inc. (Costa Mesa, CA). Affinity-purified and cross-adsorbed secondary antibodies Cy5-conjugated goat anti-mouse (H+L) was purchased from Jackson ImmunoResearch Laboratories (West Grove, PA). Rhodamine-conjugated phalloidin for actin staining were purchased from Molecular Probes (Eugene, OR).

### Immunofluorescence and epifluorescence microscopy

Cells were plated on fibronectin-coated glass coverslips for 90 min (HUVEC) to 2 hr (MEF). Cells were then permeabilized for 2 min with 0.5% Triton X-100 (Fisher Scientific) in 3% paraformaldehyde (Sigma) followed by fixation with 3% paraformaldehyde for 20 min. Antibody incubations were done for 30 min. Cells were observed on an epifluorescence Nikon TE-200 microscope. Images were captured with a Coolsnap HQ camera (Roper, Duluth, GA) with Openlab software (Improvision, Lexington, MA).

### Cell surface area analysis and kymography

Cell surface area was calculated and analyzed using Openlab software by making a binary mask from acquired images of the GFP-labeled transfected cells or the rhodamine-phalloidin-labeled non-transfected cells.

Kymography analysis of lamellipodial activity was done by using phase contrast movies of cells spreading on fibronectin-coated 35 mm glass bottom dishes (Plastek Cultureware, Ashland, MA, USA). Images were obtained at 20-second intervals for 20 minutes with the system described above. Kymography and quantitative analysis were accomplished using an original MATLAB code (Mathworks, Natick, MA).

### Analysis of Rac1 localization to focal adhesions

The percentage of focal adhesion area occupied by Rac1 wild-type or mutants was determined by using an original automation as previously described [21]. First, a binary mask corresponding to total focal adhesion area was made from the vinculin images, and the total focal adhesion area was calculated. Then, a mask corresponding to regions of Rac1 localization was made. The ratio of the two binary masks yielded the percentage of focal adhesion area showing Rac1 colocalization. Optimal spectral separation of Rac1 and vinculin was achieved by using Cy5-conjugated secondary antibody to label vinculin in cells expressing EGFP-Rac1 chimeras.

### Rac1 Binding to GTP and Western blotting

These assays were accomplished as previously described [21]. Briefly, the cDNA of the p21-binding domain (PBD) from human PAK1 (amino acids 67–150) (gift of Keith Burridge, University of North Carolina) was expressed in *E. coli* as a glutathione S-transferase fusion protein, purified, and immobilized on glutathione-sepharose beads [23]. Protein lysates from EGFP, EGFP-Rac1-wild-type- or EGFP-Rac1-mutant-transfected MEF cells were obtained two days after transfection. Cells were washed with ice-cold HEPES buffer and then lysed with lysis buffer (50 mM Tris (pH 7.6), 500 mM NaCl, 0.5 mM MgCl_2_, 1% Triton, 0.5% DOC, 0.1% SDS, 10 µg/ml leupeptin, 10 µg/ml aprotinin, 1 mM PMSF, 0.5 mM sodium vanadate). 50 µg of GST-PBD immobilized on glutathione-sepharose beads was added to approximately 800 µg of protein from cell lysates, and incubated at 4^o^C with rotation for 60 minutes. Beads were then washed, boiled in sample buffer [24], and proteins were subjected to SDS-PAGE and transferred to nitrocellulose membranes. GST-PBD bound active Rac1 (in the GTP-bound form) was detected by Western blotting using a monoclonal antibody against Rac1 (BD Transduction Labs).

Total Rac1 was detected in samples from corresponding cell lysates. Immunoblots were scanned using an Epson scanner (model 2450) and the intensity of each of the active Rac1 bands and total cellular Rac1 bands were calculated using NIH Image J software. The fraction of Rac1 that was bound to GTP, or the relative Rac1 activity was obtained by normalizing signal from the GST-PBD pull down assay to the total Rac1 band from the corresponding sample using a software-based algorithm.

### Immunoprecipitation

MEF were co-transfected with a Rac1 construct (EGFP-Rac1-WT or EGFP-Rac1-Y64F) and either Flag-βPIX, Myc-Tiam1, or Myc-RhoGDI. Two days after transfection, cells were washed with ice-cold PBS buffer and then extracted using modified RIPA buffer (0.1% DOC, 0.1% Triton X­100, 2 µM EDTA, 1 µM PMSF, 2 mM sodium vanadate, 20 µg/ml leupeptin, 20 µg/ml aprotinin in PBS). Cell lysates were clarified by centrifugation at ∼15,000 x *g* at 4^o^C for 10 minutes, and lysate volumes were normalized for equal protein content using the bicinchoninic assay (Pierce, Rockford, IL). Lysates containing 800 µg of protein were equalized for volume with lysis buffer and incubated at 4^o^C with rotation with anti-GFP antibody and then with protein A sepharose beads (Jackson) to immunoprecipitate EGFP-Rac1-WT or EGFP-Rac1-Y64F. Proteins were released from the beads by boiling in Laemmli sample buffer, subjected to SDS-PAGE using 4–15% gradient polyacrylamide gels, and transferred to nitrocellulose membranes for Western blotting. The membranes were probed with anti-GFP to determine the immunoprecipitated EGFP-Rac1 protein level and then with anti-Flag or anti-Myc to determine the differences in binding with Flag-βPIX, Myc-Tiam1, or Myc-RhoGDI.

### PAK-binding assays

GST-Rac1 fusion proteins on sepharose beads were washed once with and then resuspended in 500 µl of lysis buffer without MgCl_2_, (50 mM Tris (pH 7.6), 500 mM NaCl, 1% Triton, 0.5% DOC, 0.1% SDS, 10 µg/ml leupeptin, 10 µg/ml aprotinin, 1 mM PMSF, 0.5 mM sodium vanadate). EDTA was added to the beads slurry at a final concentration of 10 mM and mix well to dissociate nucleotide from the Rac1 fusion protein. GTPγS (0.1 mM; Sigma) was then added to the bead slurry and incubated for 30 min at room temperature with gentle shaking. A final concentration of 10 mM MgCl_2_ was added to stop the GTPγS loading process. Beads were washed three times with RIPA buffer and then added to 500 µg of HUVEC protein lysates in RIPA buffer and incubated at 4^o^C for an hour. PAK from HUVEC protein lysates that was pulled down by the GTPγS-loaded GST-Rac1 fusion proteins was revealed by immunoblotting with anti-PAK antibody. The total loading of the fusion proteins was determined by coomassie blue staining.

### In vitro kinase assays

*Escherichia coli* BL21 (Stratagene) were transformed with GST, GST-tagged Rac1-WT or Rac1-Y64F DNA constructs. GST and the GST-Rac1 fusion proteins were purified from bacterial lysates, immobilized on glutathione-sepharose beads, and then released with elution buffer (50 mM Tris, pH 8.0, 10 mM reduced glutathione; Sigma, St. Louis, MO) and quantified using Pierce Coomassie protein reagent (Fisher Scientific, Hampton, NH). Approximately 5 µg of GST, GST-Rac1-WT or GST-Rac1-Y64F were incubated with differential amounts of purified, baculovirus-derived Src (aa 86-536), or GST-tagged wild-type FAK (GST-wtFAK411-686) in the presence or absence of 20 µM ATP in kinase buffer (50 mM Tris, pH 7.4, 5 mM MnCl_2_, and 5 mM MgCl_2_). The total volume of each reaction was adjusted to 60 µl and samples were incubated at 37^o^C for 30 min with occasional shaking to keep the beads in suspension. The kinase reactions were stopped by the addition of sample buffer [24], analyzed using SDS-PAGE, transferred to nitrocellulose, and serially immunoblotted with antibodies against GST, Rac1, Src or FAK, and phosphotyrosine.

### Statistical Analysis

The student t-test (two-tailed) was used to analyze cell surface area, the percentage of the focal adhesion area occupied by Rac1, Rac1 binding to GTP, and Rac1 binding to Flag-βPIX, Myc-Tiam1 and Myc-RhoGDI. P values are supplied in each figure legend, and significance was adjudged to be present at p values less than 0.05 for all data. All graphs include standard error bars.

## Results

### Cells expressing Rac1-Y64D and Rac1-Y64F exhibit changes in spreading and lamellipodial dynamics

The present study was motivated by our previously findings that FAK-mediated tyrosine phosphorylation of βPIX enhanced both guanine nucleotide exchange on Rac1 and cell spreading [21], together with findings of Tu and Cerione on the effects of tyrosine phosphorylation of Cdc42 at Y64 and the sequence identity between Cdc42 and Rac1 at this region of Rac1 (Figure 1). We therefore studied cell spreading and membrane extension kinetics in cells expressing Rac1 mutations that either obviate tyrosine phosphorylation at Y64 or mimic constitutive phosphorylation at this position. The hypothesis at this stage was that tyrosine phosphorylation would augment Rac1 activity and membrane extension. A site-directed mutagenesis strategy was used to produce Y64F and Y64D mutations in EGFP-Rac1.

**Figure 2:**
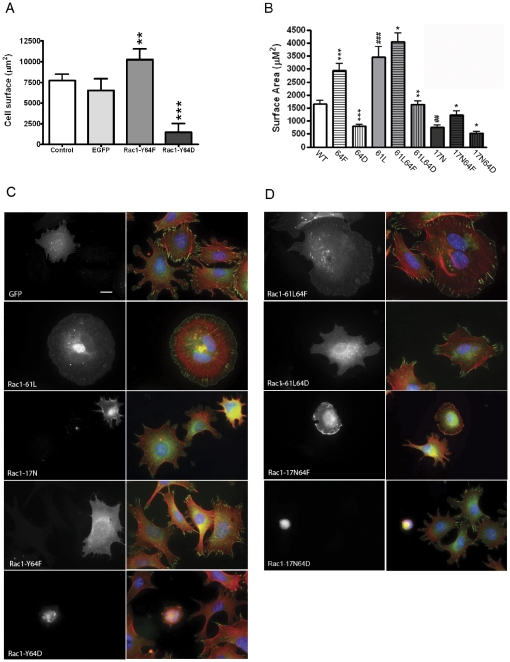
Rac1-Y64 phosphorylation modulated cell spreading. (A) Transfected HUVEC were plated onto fibronectin-coated coverslips for 90 minutes and then fixed and permeabilized for immunofluorescence staining. Rhodamine-phalloidin labeled EGFP-positive cells were used for cell surface area quantification using Openlab software. Data are shown as mean±SEM, and were as follows: non-transfected HUVEC controls had a mean surface area of 7760 µm^2^ (± 730.5, n = 28), and the mean area was 6510 µm^2^ (±1433.0, n = 8) for EGFP-transfected cells. A large increase in spread surface area to 10246 µm^2^ (±1310, n = 14) was noted in EGFP-Rac1-Y64F-transfected HUVEC, and cells expressing EGFP-Rac1-Y64D had a greatly reduced mean surface area of 1480 µm^2^ (±1037, n = 5). ** indicates p <0.001, and *** denotes p < 0.0001 for the designated population as compared with the EGFP control. (B) Cell surface area was quantified from phalloidin-labeled MEF images using Openlab software in cells transfected with EGFP-Rac1-WT, EGFP-Rac1-Y64F, EGFP-Rac1-Y64D, EGFP-Rac1-61L, EGFP-Rac1-61L/64F, EGFP-Rac1-61L/64D, EGFP-Rac1-17N, EGFP-Rac1-17N/64F, or EGFP-Rac1-17N/64D. Data are shown as the mean±SEM. * denotes p < 0.05, ** means p <0.001, and *** is p < 0.0001 when data from groups transfected with Y64F or Y64D are compared with values from the corresponding controls (e.g. EGFP-Rac1-WT, EGFP-61L, or EGFP-17N, respectively, without the mutations at position 64). ## denotes p<0.005, while ### denotes p<0.0001 when data from 61L or 17N mutants were compared with wild-type. Quantitative cell spreading data and the number of MEF studied in each group are detailed in Table 1. (C) MEF were transfected with EGFP, or EGFP-tagged Rac1 single mutants. Two days after transfection, cells were seeded onto fibronectin-coated coverslips for two hours and prepared for immunofluorescence analysis. Cells were labeled with DAPI, rhodamine-phalloidin and anti-vinculin (followed by Cy5). Transfected GFP-positive cells are shown indicated in black and white, while the composite images display merges of combined DAPI (blue), phalloidin (red) and vinculin (green) labeling. Scale bar denotes 20 µm. (D) MEF that had been transfected with the EGFP-tagged Rac1 double mutants including Rac1-61L/64F, -61L/64D, -17N/64F, or -17N/64D were plated on fibronectin-coated coverslips and labeled for IF analysis as describe for panel 2C. Scale is the same as panel 2C.

Consequences of these mutations were studied in HUVEC (Figure 2A). After 90 min of adhesion to fibronectin-coated glass coverslips, cells were fixed and prepared for epifluorescence analysis. Surface area data were calculated from 2-D images of paraformaldehyde-fixed cells using an algorithm in Openlab software after labeling with rhodamine-conjugated phalloidin. EGFP-transfected cells did not spread differently than control non-transfected HUVEC at this time point. Strikingly, in HUVEC transfected with EGFP-Rac1-Y64D that mimics a constitutively phosphorylated tyrosine at position 64, the mean surface area decreased markedly. Also of note was the large increase in average cell surface area at 90 min over controls in EGFP-Rac1-Y64F-transfected HUVEC.

**Table 1:** Rac1 Mutations at Y64 Induced Changes in MEF Spreading.

Transfected Construct	Number of Cells	Mean Surface Area (µm^2^)	Standard Error
EGFP	38	1666	133.7
61L	28	3448	422.2
17N	18	751.8	88.79
64F	26	2930	290.1
64D	32	791.8	80.03
61L/64F	23	4052	324.3
61L/64D	20	1627	143.6
17N/64F	21	1229	152.84
17N/64D	34	523.4	79.68

MEF expressing these mutants or Rac1-61L or Rac1-17N were analyzed for spreading after seeding on FN-coated coverslips for 2 hr was determined by using the method described above, and data are shown in Figure 2B and 2C. Numerical results and sample sizes for each experimental group are detailed in Table 1. Expression of EGFP-Rac1-Y64D reduced spreading by over 50% as compared with MEF expressing EGFP-Rac1-WT, while EGFP-Rac1-Y64F expression increased spread cell surface area by 75% of the control value.

To further investigate the interaction of phosphotyrosine-based regulation of Rac1 function with the biology of constitutively active (61L) or dominant negative (17N) changes in Rac1 function, four additional constructs were produced with double mutations - EGFP-Rac1-61L/64F, EGFP-Rac1-61L/64D, EGFP-Rac1-17N/64F, and EGFP-Rac1-17N/64D. These data are shown in Figure 2 B and D, and details are tabulated in Table 1. Rac1-61L-induced MEF spreading was inhibited in cells expressing the double mutant 61L/64D, but increased in MEF expressing 61L/64F. In the case of MEF that were expressing EGFP-Rac1-17N, double mutant 17N/64D expression caused a further decrement in cell spreading as compared with cells expressing Rac1-17N, whereas transfectants expressing EGFP-Rac1-17N/64F exhibited partial rescue of cell spreading function as compared with MEF expressing Rac1-17N alone. Thus, the addition of either the Y64D or the Y64F mutations made a statistically significant impact in the spreading behavior of MEF expressing either Rac1-61L or Rac1-17N. These data indicated that tyrosine phosphorylation at Y64 may be a negative regulatory input for membrane extension that has a separate mechanism from the status of GTP-binding.

MEF expressing EGFP-Rac1-Y64D were rounded and not well flattened. They had focal adhesions around the cell periphery but not focal complexes or well-developed lamellipodia (Figure 2C). On the contrary, EGFP-Rac1-Y64F, established broad curvilinear lamellipodia and prominent peripheral focal complexes. The enhanced cell spreading noted in MEF that were expressing EGFP-Rac1-61L was further augmented by the addition of the Y64F mutation without dramatic effect on focal adhesion or focal complex morphology. The addition of the Y64D mutation, on the other hand, reduced focal complex number, peripheral focal adhesions, and cell spreading in both the Rac1-61L and the Rac1-17N backgrounds. The absence of apoptosis in MEF expressing EGFP-Rac1-Y64D was shown by the absence of caspase 3 cleavage in all 9 of the cells studied (Figure S1). In contrast, all 11 of the H_2_O_2_-treated MEF studied were positive for cleaved caspase 3.

**Figure 3:**
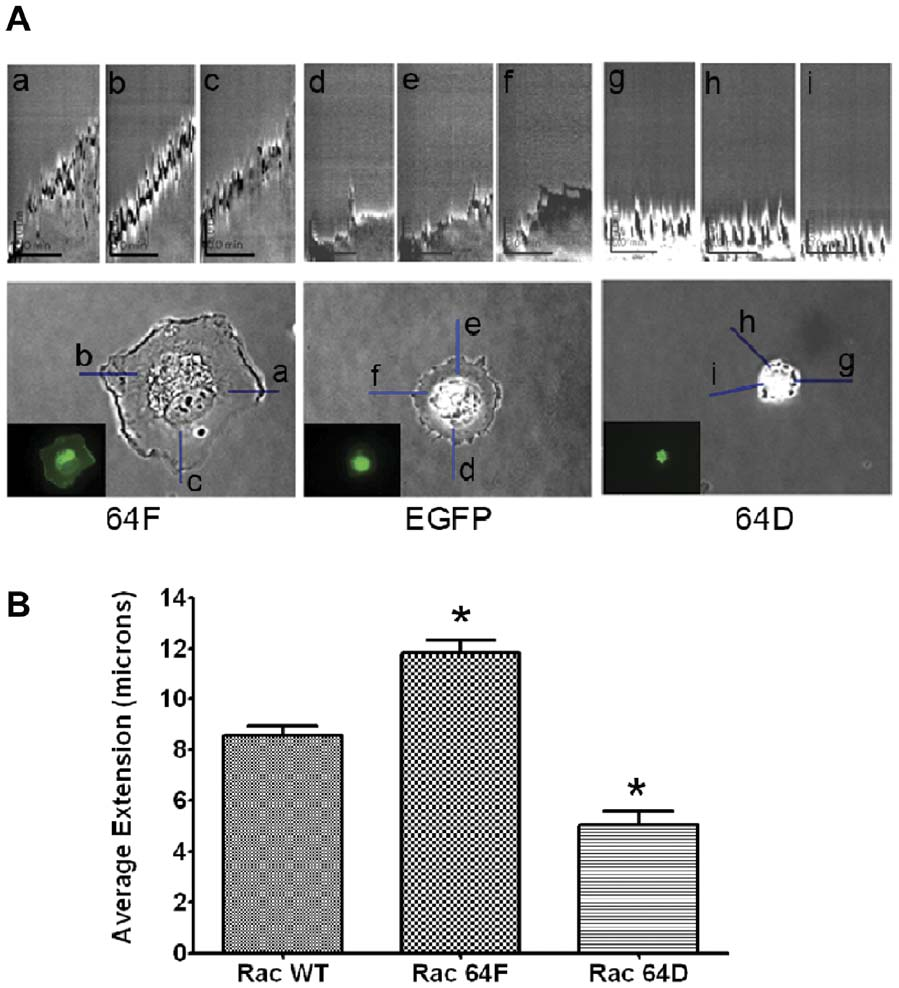
Expression of Rac1-Y64F or Rac1-Y64D altered lamellipodial dynamics. HUVEC cells were transfected with EGFP, or EGFP-tagged Rac1-Y64D, or Rac1-Y64F. Cells were plated onto fibronectin-coated coverslips two days after transfection, and were observed under phase contrast microscopy until initial attachment was achieved (typically 10 minutes). Images were taken every 20 seconds for 20 minutes. Kymograph analysis of cell spreading efficiency was performed using these serial images. (A) Three sets of kymographs are shown from each transfected cell: a–c for EGFP-Rac1-Y64F; d-f for EGFP and g-i for EGFP-Rac1-Y64D. The lower panel shows the transfected cells that were studied with the line scan used for kymograph. The inset shows the GFP signal of the transfected cells. (B) Kymography from three to six transfected cells were analyzed to study the differences in their spreading. Significant differences in lamellipodial extension were noted in both cells expressing Rac1-Y64F and Rac1-Y64D when compared with HUVEC expressing Rac1-WT (p values  =  0.0377 and 0.0005 respectively).

Kymography was then used to study the rate of membrane extension and lamellipodial persistence in HUVEC that were expressing EGFP, EGFP-Rac1-Y64D or EGFP–Rac1-Y64F (Figure 3)[25]. Forty-eight hours following transfection, HUVEC cells were plated on FN-coated 35-mm glass bottom dishes for 10 minutes, and then studied by phase contrast microscopy at 20 seconds intervals for 20 minutes. Line scans corresponding to the presented kymographs are shown on the phase image in Figure 3A. These studies revealed two key insights. First, expression of the Rac1-Y64F mutation was associated with an increase in the rate of membrane extension as compared to the control, and intermittent retraction of the lamellipodial edge seemed to occur less frequently. An average membrane extension rate of 0.934 µm/min was observed for Rac1-Y64F-transfected cells, as compared with 0.294 µm/min for EGFP-transfected HUVEC. In the Rac1-Y64D-transfected cells, the rate of membrane extension was only 0.039 µm/min. Second, the leading edges of HUVEC expressing EGFP-Rac1-Y64D were more active than the wild type controls, and demonstrated very rapid membrane movements. These were characterized by short-lived protrusions that were quickly and almost completely retracted, and a failure of these cells to establish and maintain consistent lamellipodia. On average, there was one cycle of brief rapid extension followed by quick retraction every 2.5 min in the cells expressing Rac1-Y64D. This pattern of extension and retraction of the leading membrane edge was also observed in the EGFP-transfected control cells within the first 5 min of plating, but this behavior quickly disappeared and stable lamellipodia were then established. In contrast, EGFP-Rac1-Y64D-transfected cells continued to exhibit this pattern of rapid and extension and retraction with little spreading at 60 minutes following plating. In Rac1-Y64F-transfected cells, stable lamellipodial extension began immediately after cell attachment to matrix. Statistical analysis of multiple cells and kymographs for Rac1-WT, -64F, or -64D transfectants are shown in Figure 3B, and additional images are shown for each cell population in Figure S2. These findings suggest a role for tyrosine phosphorylation in the modulation of Rac1 function during the stabilization of cytoskeletal infrastructure and/or transmembrane adhesions during cell spreading and movement.

### Focal Adhesion targeting of Rac1 is modulated by Y64 phosphorylation

**Figure 4:**
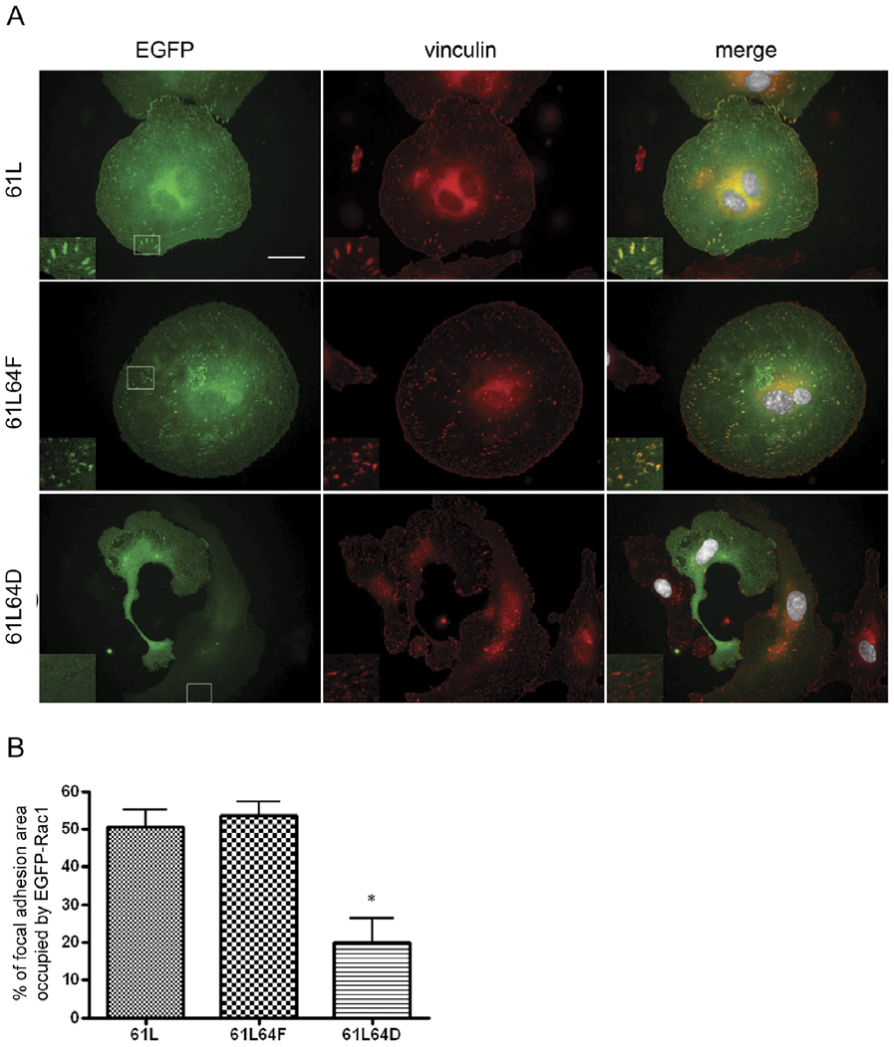
Y64 phosphorylation modulated focal adhesion targeting of active Rac1. (A) HUVEC cells were transfected with EGFP-tagged Rac1 mutants including Rac1-61L, Rac1-61L64F and Rac1-61L64D. Cells were labeled with DAPI and anti-vinculin (followed by Cy5). Transfected EGFP-positive cells are shown in green, while the vinculin labeled focal adhesions shown in red. The composite images display merges of combined GFP (green), DAPI (white), and vinculin (red) labeling. The boxed areas were magnified (2X) and shown in the insets. Scale bar denotes 30 µm. (B) The percentage of the total focal adhesion area (defined by vinculin labeling) that was occupied by EGFP-tagged Rac1 mutants in transfected cells were quantified using Openlab software. Fifteen randomly picked transfected cells were analyzed in each group, and data are shown as the mean±SD. * denotes p < 0.05 when data from groups transfected with 61L/64F or 61L/64D are compared with values from the group transfected with 61L alone.

Our previous work documented activity-dependent targeting of Rac1 to focal adhesions [21]. In order to investigate the effects of Y64 phosphorylation on the subcellular targeting of active Rac1, the spatial localization of EGFP-Rac1-61L, EGFP-Rac1-61L/64F and EGFP-Rac-61L/64D were analyzed in transfected HUVEC (Figure 4, A and B). After 90 minutes of adhesion to fibronectin-coated glass coverslips, both EGFP-Rac1-61L and EGFP-Rac1-61L/64F exhibited a high degree of targeting to vinculin-containing focal adhesions, with average values of 50.56±15.01% and 53.70%±11.03% respectively. However, the activation-driven effects of the 61L state on Rac1 targeting was apparently antagonized by the simultaneous expression of Y64D, such that only 20.19±19.39 % of focal adhesion area was occupied by EGFP-Rac1-61L/64D. These data suggest a role for tyrosine phosphorylation at Rac1-Y64 in the regulation of Rac trafficking to matrix adhesion sites.

### Mutations at Rac1-Y64 change binding and association with GTP, GEFs, PAK, and RhoGDI

**Figure 5:**
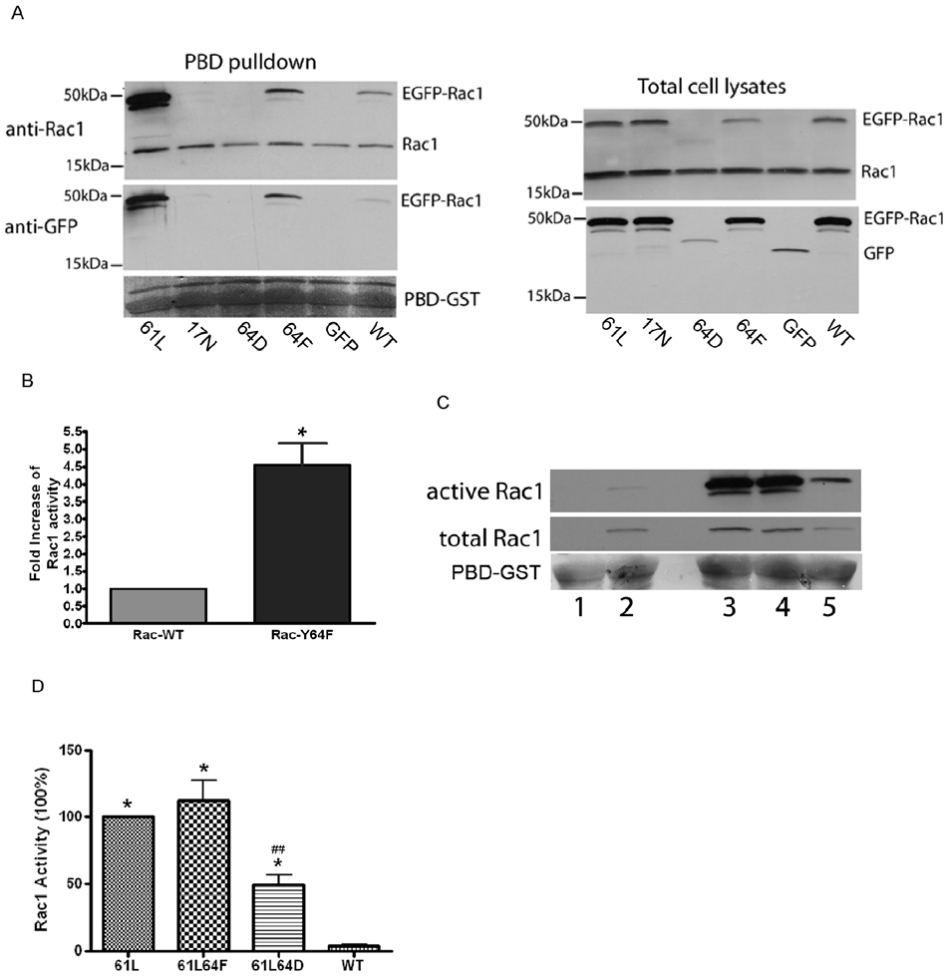
Mutations in Rac1-Y64 changed binding to GTP. (A) Left panel: MEF cells were transfected with Rac1 mutants including EGFP-Rac1-61L (lane 1 EGFP-Rac1-17N (lane 2), EGFP-Rac1-Y64D (lane 3), or EGFP-Rac1-Y64F (lane 4), or with EGFP (lane 5), or EGFP-Rac1-WT (lane 6). Coomassie blue staining of the membrane (bottom row, left) verified the addition of an equal amount of PBD-GST. Western blotting was done using both anti-Rac1 and anti-GFP antibodies. Right panel: 15 µg of total protein lysates were also analyzed by SDS-PAGE and immunoblotted to verify equal loading and the expression of the transfected EGFP-Rac1 constructs. (B) Four sets of Rac1 activity assay results were used for densitometry and statistical analysis of the difference in activity between Rac1-WT and Rac1-Y64F. Western blots were scanned and Rac1 activity was normalized to the total cellular Rac1. Data shown are mean values±SEM. * denotes p < 0.02. (C) Protein lysates were harvested from MEF (lane 1) or MEF that were transfected with EGFP-Rac1WT (lane 2), EGFP-Rac1-61L (lane 3), EGFP-Rac1-61L/64F (lane 4), or EGFP-Rac1-61L/64D (lane 5). PBD-GST pull down assays were performed with 800 µg of total protein to determine Rac1 activity. The membrane was immunoblotted with anti-Rac1 to demonstrate the level of Rac1 activity, and was then stained with Coomassie blue to ascertain the relative amounts of PBD-GST substrate added to each sample. Expression efficiency of the wild type and mutant EGFP-Rac1 constructs was assayed using 15 µg of total protein from whole cell lysates and the Rac1 activity was normalized to the respective protein level of each sample. (D) Five sets of experimental data were analyzed to examine the differences between the Rac1 activity in lysates from MEF expressing EGFP-RacWT, EGFP-Rac1-61L, EGFP-Rac1-61L/64F, and EGFP-Rac1-61L/64D. ECL blots were scanned and analyzed using Image J software. The intensity of each of the active Rac1 bands pulled down by PBD was normalized to the total cellular Rac protein band and calculated by a software-based algorithm. The Rac1 activity in lysates from cells expressing EGFP-Rac1-61L was assigned a relative value of 100%. ## indicated a significant decrease of Rac1 activity in 61L/64D compared with 61L (p < 0.01). * indicated a significant increase in Rac1 activity in 61L, 61L/64F and 61L/64D compared with Rac1 wild-type (p > 0.001).

To further elucidate the molecular mechanisms of Rac1-Y64F enhancement of cell spreading, lamellipodial stability, and the targeting of Rac1 to focal adhesions, we studied Rac1 activity (e.g. GTP binding and not GTPase activity) in MEF that were transfected with EGFP-Rac1-WT, or mutations that included Q61L, T17N, Y64D, or Y64F (Figure 5). Rac1 activity was analyzed in aliquots of lysates from transfected MEF by a GST affinity pull-down assay using the Rac-binding domain of PAK and 15 µg of protein from each sample were blotted with anti-Rac1 antibody for total Rac1 as a loading control [21]. MEF expressed fairly comparable amounts of the respective EGFP-tagged Rac1 proteins, wild type or mutants, except for EGFP-Rac1-Y64D, which appeared to be quickly degraded. When 800 µg of total protein lysates were reacted *in vitro* with 50 µg of GST-PBD produced in *E. coli*, the controls were as expected: more amount of the constitutively active Rac1-61L was pulled down as compared to the wild type, and no dominant negative Rac1-17N was pulled down. Interestingly, more of the EGFP-Rac1-Y64F was pulled down by GST-PBD than the wild type Rac1, indicating more GTP binding and higher Rac1 activity in the presence of the Y64F mutation (Figure 5A). The degraded EGFP-Rac1-Y64D product did not bind to GST-PBD indicating a decrease in both GTP binding and Rac1 activity. Densitometry data from four sets of these experiments are shown in Figure 5B. GTP-bound active Rac1 in the pull downs was normalized to the amount found by Western blot in total lysates. A 3.5 fold increase in Rac1 activity was observed for the EGFP-Rac1-Y64F mutant compared to the wild type. Interestingly, in the background of the constitutively active 61L mutation, 64F and 64D mutations also affected Rac1 activity. Unlike the single mutation EGFP-Rac1-Y64D which was degraded in transfected cells, EGFP-Rac1-61L/64D was more stable. EGFP-Rac1-61L/64D had lower Rac1 activity than either the 61L or 61L/64F mutants (Figure 5C and D).

**Figure 6:**
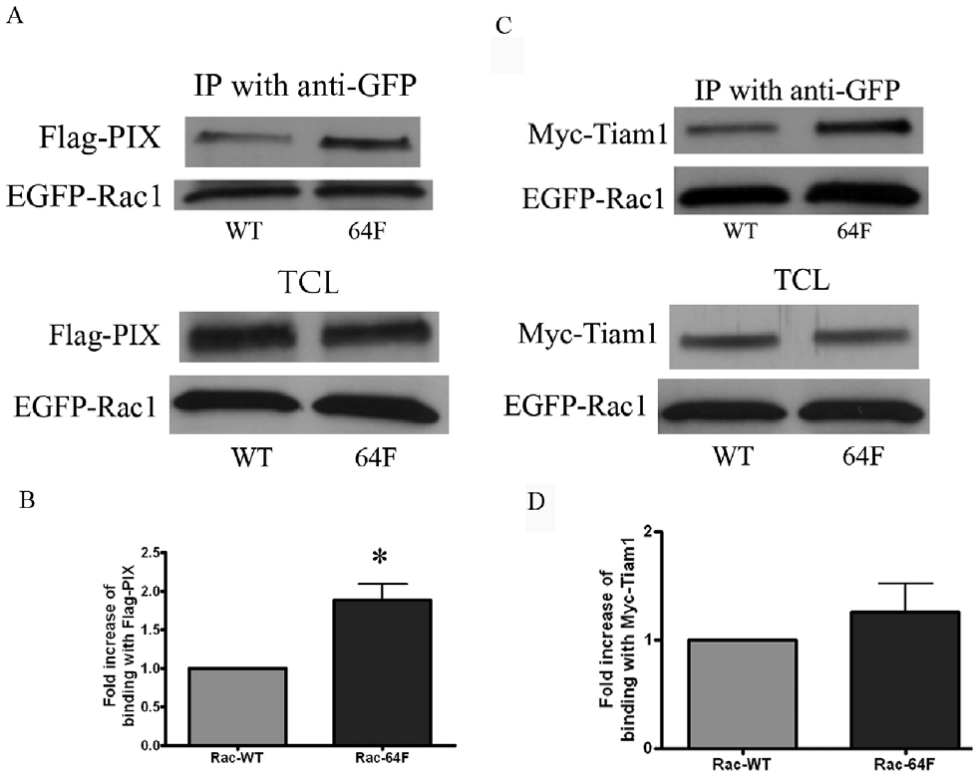
The Y64F mutation in Rac1 changed binding to guanine nucleotide exchange factors. (A) Protein lysates were harvested from MEF that were co-transfected with Flag-tagged βPIX, and either EGFP-tagged Rac1WT (lane 1) or Rac1-Y64F (lane 2). Ectopically expressed EGFP-Rac1 proteins, wild-type or Y64F mutant, were immunoprecipitated with anti-GFP antibody and the membrane was blotted with anti GFP to demonstrate the expression level of EGFP-Rac1 proteins and also with anti-Flag antibody to reveal the Flag-βPIX that was co-immunoprecipitated with EGFP-Rac1 proteins. Immunoblotting of total cell lysates (TCL) for Flag-βPIX and EGFP-Rac1 are shown below the co-immunoprecipitation study as controls. (B) Four sets of experimental data were analyzed for the differences in binding to Flag-tagged βPIX for EGFP-Rac1WT or EGFP-Rac1-Y64F. The intensity of the co-immunoprecipitated Flag-βPIX was normalized to the EGFP-Rac1 pulled down by anti-GFP antibody. The amount of Flag-βPIX co-immunoprecipitated with EGFP-Rac1WT was assigned a relative value of 100%. EGFP-Rac1-Y64F bound more efficiently to Flag-βPIX (p  =  0.0223). (C) Protein lysates were harvested from MEF that were co-transfected with Myc-tagged Tiam1 and either EGFP-Rac1WT (lane 1) or EGFP-Rac1-Y64F (lane 2). Ectopically expressed EGFP-Rac1 proteins were immunoprecipitated with anti-GFP antibody and the membrane was blotted with anti GFP to demonstrate the expression level of EGFP-Rac1 proteins, and with anti-Myc antibody to reveal the Myc-Tiam1 that was co-immunoprecipitated with the EGFP-Rac1 constructs. Immunoblotting of total cell lysates (TCL) for Myc-Tiam1 and EGFP-Rac1 are shown below the co-immunoprecipitation study as controls. (D) Six sets of experimental data were analyzed for differences in binding to Myc-Tiam1 between EGFP-Rac1WT and EGFP-Rac1-Y64F. The intensity of the immunoprecipitated Myc-Tiam1 was normalized to the EGFP-Rac1 pulled down by anti-GFP antibody. The amount of Myc-Tiam1 co-immunoprecipitated with EGFP-Rac1WT was assigned a relative value of 100%. The difference did not reach statistical significance p  =  0.3793).

We next investigated the possibility that mutations at Y64 affected the association of Rac1 with two Rac1-associated GEF proteins that can perform nucleotide exchange on Rac1 - βPIX and Tiam1 (Figure 6). MEF cells were co-transfected with either EGFP-Rac1-WT or EGFP-Rac1-Y64F, and with either Flag-tagged βPIX or Myc-tagged Tiam1. The EGFP-Rac1 constructs were immunoprecipitated with anti-GFP antibody and immunoblotting with anti-Flag or anti-Myc antibodies was used to interrogate the amounts of Flag-βPIX or Myc-Tiam1 that bound to the respective EGFP-Rac1 proteins. The Y64F mutant doubled Rac1 interaction with βPIX (Figure 6, A and B), but the data on Tiam1 association revealed an increase in association with EGFP-Rac1-Y64F as compared with the wild type EGFP-Rac1 that did not reach statistical significance (Figure 6, C and D). These data suggest that increased association with GEFs may contribute to increased GTP loading on Rac1-Y64F, and to the increased spreading seen in cells expressing this construct.

**Figure 7:**
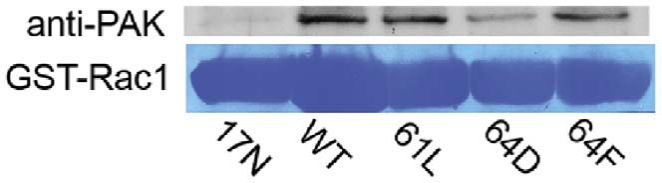
The Rac1-64D mutation caused decreased Rac1 binding to PAK. 30 µg of purified GST-tagged Rac1 17N (lane 1), wild-type (lane 2), 61L (lane 3), 64D (lane 4) or 64F (lane 5) were bound to sepharose beads and loaded with GTPγS for 30 min before incubation with 400 µg of HUVEC protein lysates in order to assay PAK binding. Unlike the 17N mutant which can not bind GTP, Rac1-64D does bind GTP and pull down PAK. However, PAK binding by this mutant is reduced compared to that seen for Rac1-WT, -61L, or -64F.

To test the impact of Rac-1-Y64D or Y64F expression on Rac1 interaction with its molecular effectors, PAK-binding assays were performed (Figure 7). Purified GST-tagged Rac1-WT, Rac1-17N, and the previously detailed mutants were isolated on sepharose, loaded with non-hydrolyzable GTPγS, and incubated with native PAK from HUVEC lysates. Rac1-17N did not bind to PAK, whereas Rac1-64D exhibited decreased binding compared with Rac1-WT, -61L, or -64F. This finding indicates that phosphorylation of tyrosine at position 64 in Rac1 may downregulate, but not abrogate, Rac1 binding to both GTP and PAK.

**Figure 8:**
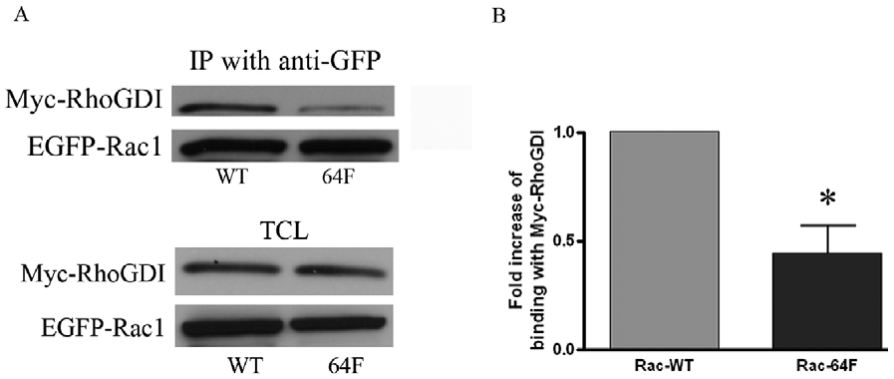
The Y64F mutation in Rac1 decreased binding to RhoGDI. (A) Protein lysates were harvested from MEF that were co-transfected with Myc-tagged RhoGDI and either EGFP-tagged Rac1WT (lane 1) or Rac1-Y64F (lane 2). EGFP-Rac1WT or EGFP-Rac1-Y64F proteins were immunoprecipitated with anti-GFP antibody and the membrane was blotted first with anti-GFP to demonstrate the expression level of EGFP-Rac1 proteins, and then with anti-Myc antibody to show the Myc-RhoGDI that co-immunoprecipitated with EGFP-Rac1 proteins. Immunoblotting of total cell lysates (TCL) for Myc-RhoGDI and EGFP-Rac1 are shown below the co-immunoprecipitation study as controls. (B) Five sets of experimental data were analyzed for the differences between EGFP-Rac1WT and EGFP-Rac1-Y64F interaction with Myc-RhoGDI. ECL blots were scanned, normalized, and analyzed as described above. Myc-RhoGDI bound less well to EGFP-Rac1-Y64F as compared to EGFP-Rac1WT (p  =  0.011).

Finally, since RhoGDI binding may regulate subcellular trafficking, sequestration and interactions with downstream substrates for Rho family GTP binding proteins, we examined RhoGDI-binding in EGFP-Rac1-Y64F as contrasted with wild-type Rac1 [11]. A representative study and a compilation of five sets of data from these experiments are shown in Figure 8. A greater than 50% drop in RhoGDI binding was associated with expression of the Y64F mutation in EGFP-Rac1.

### Both Src and FAK phosphorylate Rac1 at tyrosine 64

**Figure 9:**
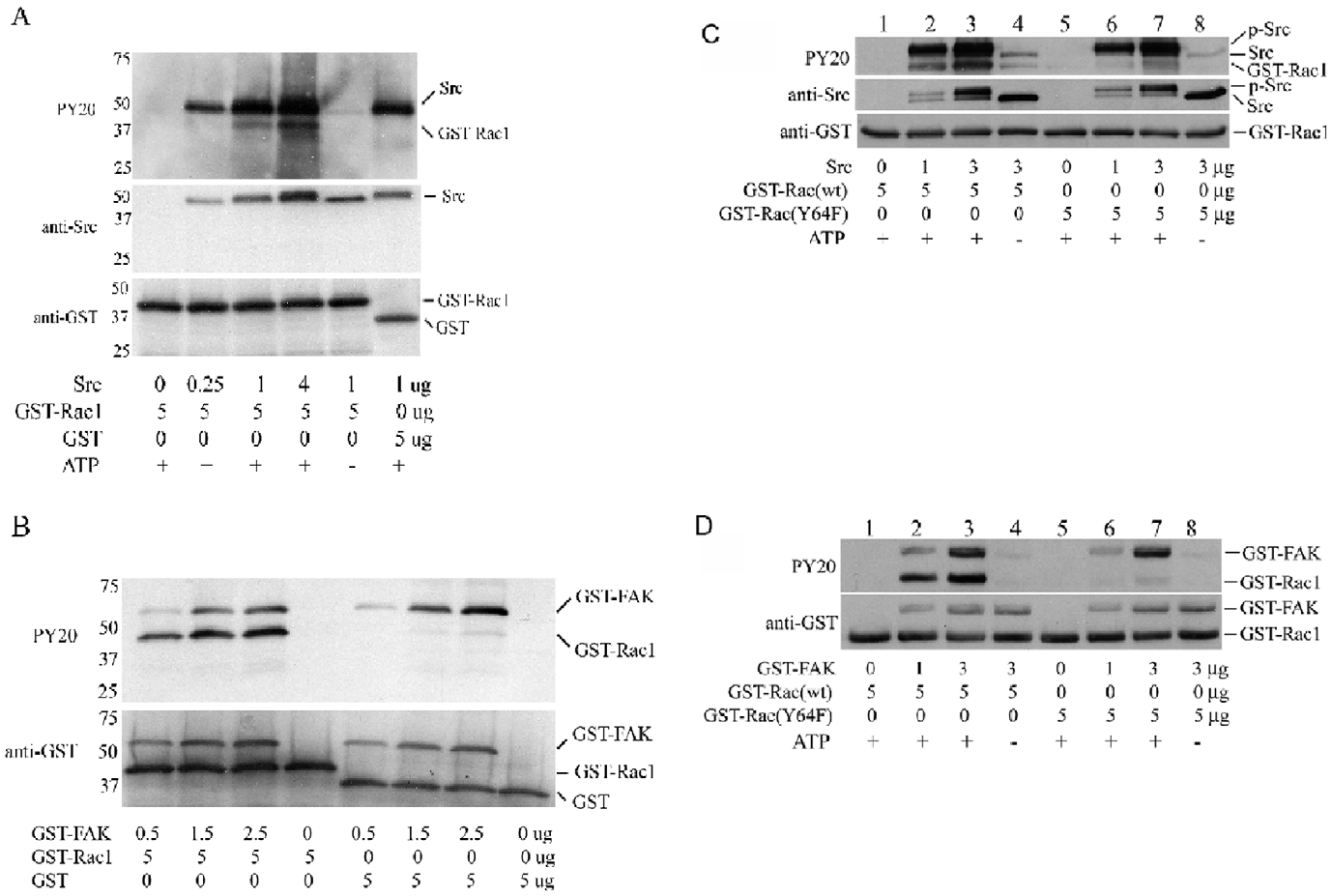
Src and FAK mediated tyrosine phosphorylation of Rac1 at tyrosine 64 in vitro. (A) 5 µg of either GST-Rac1 (lanes 1–5) or GST (lane 6) were incubated with 0 µg (lane1), 0.25 µg (lane 2), 1 µg (lanes 3, 5, 6), or 4 µg (lane 4) of purified Src in kinase buffer. All samples included 20 µM ATP except for the one shown in lane 5. The kinase reaction mixture was analyzed by SDS-PAGE and immunoblotted with antibodies against phosphotyrosine, Src, and GST. (B) 5 µg of GST-Rac1 (lanes 1–4) or GST (lanes 5–8) were incubated with 0 µg (lanes 4, 8), 0.5 µg (lanes 1, 5), 1.5 µg (lanes 2, 6), or 2.5 µg (lanes 3, 7) of GST-tagged wild-type FAK (GST-wtFAK411-686) in kinase buffer with 20 µM ATP. The kinase reaction mixture was analyzed by SDS-PAGE, and immunoblotted with antibodies against phosphotyrosine and GST. (C) 5 µg of GST-Rac1-WT (lanes 1–4) or GST-Rac1-Y64F (lanes 5–8) were incubated with 0 µg (lanes 1, 5), 1 µg (lanes 2, 6), or 3 µg (lanes 3, 4, 7, 8) of purified Src. Reactions run in all lanes contained 20 µM ATP, except for lanes 4 and 8. The kinase reaction mixture was analyzed by SDS-PAGE and immunoblotted with antibodies against phosphotyrosine, Src, and GST. (D) 5 µg of GST-Rac1-WT (lanes 1–4) or GST-Rac1-Y64F (lanes 5–8) were incubated with purified GST-WT-FAK (aa 411-686) in the following amounts: 0 µg (lanes 1, 5), 1 µg (lanes 2, 6), or 3 µg (lanes 3, 4, 7, 8). All reactions included 20 µM ATP except for the ones represented in lanes 4 and 8. The kinase reaction mixture was analyzed by SDS-PAGE and immunoblotted with antibodies against phosphotyrosine and GST.

Src and FAK have been shown to function cooperatively in the tyrosine phosphorylation of downstream substrate proteins during integrin signaling [26]–[30]. In order to ascertain whether Src and FAK could separately and directly tyrosine phosphorylate Rac1, in vitro kinase assays were performed (Figure 9A and B). Five µg of purified GST-Rac1 were incubated with increasing amounts of human Src (aa 86-536) (0, 0.25, 1 and 4 µg) or GST-FAK (aa 411–686) (0, 0.5, 1.5 and 2.5 µg) in kinase buffer with or without ATP (for the Src assay). Kinase reaction mixtures were then separated by SDS-PAGE and blotted with anti-Src, anti-GST, and anti-phosphotyrosine to determine the kinase activity of Src and FAK on Rac1 tyrosine phosphorylation (Figure 9). Five µg of GST were used as a control substrate. The results demonstrated that specific, dose-dependent phosphorylation of GST-Rac1 was mediated by both Src and FAK. The absence of tyrosine phosphorylation when GST was used as substrate indicated that Rac1, and not the GST tag, was the phosphorylation target.

Src has previously been shown to phosphorylate Cdc42 on tyrosine 64, and pY64-Cdc42 showed stronger binding affinity to RhoGDI than non-phosphorylated Cdc42 [20]. Analysis with the phosphorylation site module of the prediction program PHOSIDA (www.phosida.com) [31] identified Y64 as one of the two major candidate phosphorylation sites on Rac1. Therefore, Tyr64 was changed to Phe64 using site-directed mutagenesis in order to study the role of this residue in the process of Rac1 phosphorylation by Src or FAK *in vitro*. Five µg of purified GST-Rac1 or GST-Rac1-Y64F were incubated with 0, 1, or 3 µg of purified Src or GST-FAK in kinase buffer with or without ATP. Resulting kinase reaction mixtures were subjected to SDS-PAGE and immunoblotted with antibodies against Src, GST, and phosphotyrosine (Figure 9C and D). This analysis demonstrated equal loading of GST-Rac1 and GST-Rac1-Y64F in the kinase reaction mixtures (lower panel of Figure 9C and D; FAK used in this kinase assay was also tagged with a GST, and thus revealed in the GST blot). Anti-Src immunoblots showed a doublet (middle panel in Figure 9C) - the upper band of the doublet is tyrosine-phosphorylated Src and the lower band of the doublet is non-tyrosine-phosphorylated Src. Dose-dependent, specific tyrosine phosphorylation of GST-Rac1 that required ATP was seen with both Src and FAK, but tyrosine phosphorylation by either Src or FAK was all but abolished on GST-Rac1-Y64F (Figure 9, C and D). These data indicate that a specific, dose-dependent tyrosine phosphorylation of Rac1 at Y64 by both FAK and Src may occur *in vitro*.

### Src mediates tyrosine phosphorylation of Rac1 in MEF

**Figure 10:**
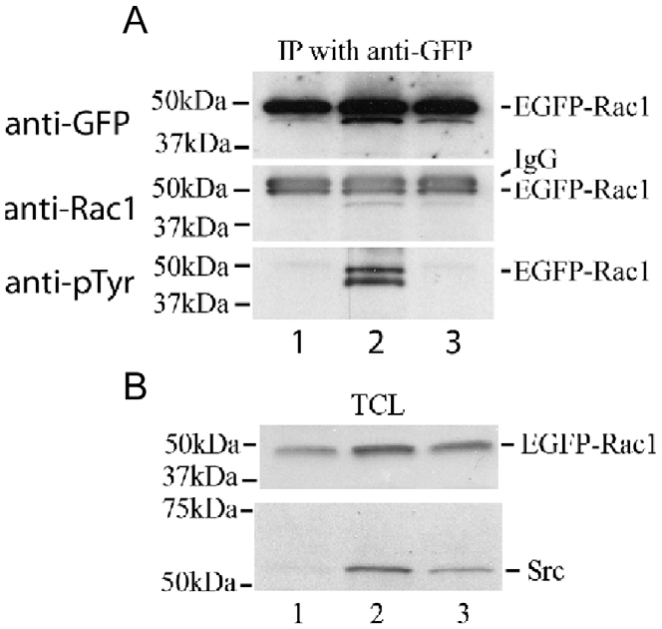
Src tyrosine-phosphorylated Rac1 in MEF. (A) SYF cells were transfected with EGFP- Rac1-WT alone (lane 1) or together with wild-type Src (lane 2) or kinase dead Src (lane 3). EGFP-Rac1 immunoprecipitates were obtained with anti-GFP antibody two days after transfection, and immunoblotted with anti-GFP and anti-Rac1antibodies to quantify EGFP-Rac1 expression, and then with anti-phosphotyrosine to probe for tyrosine phosphorylation of EGFP-Rac1, as seen in cells that were transfected with exogenous Src. Molecular weight markers are shown in kilodaltons (kDa) on the left. (B) Immunoblotting of total cell lysates (TCL) for EGFP-Rac1 and Src are shown as a control.

To test whether Src can specifically tyrosine-phosphorylate Rac1 *in vivo*, SYF (triple knock out) cells were transfected with EGFP-tagged wild-type Rac1 alone, or together with either wild-type Src or kinase dead Src. Ectopically expressed EGFP-Rac1 was immunoprecipitated with anti-GFP antibody and blotted with anti-phosphotyrosine antibody to determine the tyrosine phosphorylation level of the EGFP-Rac1. Immunoblotting of these same membranes with anti-GFP or anti-Rac1 antibodies provided a measure of the expression level of EGFP-Rac1. Our results showed that expression of the full length EGFP-Rac1 (∼48.4 kDa) was not affected by singular transfection or double transfection with Src. Interestingly the EGFP-Rac1 when transfected alone or with a kinase-dead Src was not tyrosine phosphorylated. Only when SYF cells were co-transfected with wild-type Src, was the EGFP-Rac1 phosphorylated on tyrosine (Figure 10).

## Discussion

Tyrosine phosphorylation events drive many essential events in the life of eukaryotic cells, and the nonreceptor tyrosine kinases FAK and Src have diverse roles in developmental, vascular, and cancer biology [26], [30], [32]–[36]. The current investigations contribute additional facets to the understanding of phosphotyrosine signaling and identify a new possible regulatory input in the modulation of Rac1, a key molecular maestro in the coordination of cytoskeletal responses to the cellular microenvironment. The data presented here suggest structure-function relationships within the Rac1 Switch II domain that may be altered by tyrosine phosphorylation and change Rac1-mediated cytoskeletal dynamics during cell spreading.

Tyrosine 64 is located in the Switch II domain of Rac1 - one of two regions that are distinguished by conformational differences between the GDP-bound (inactive) and the GTP-bound (active Rac1) states [37]. The conformational sensitivity of this location and its susceptibility to phosphorylation strongly suggest its importance in regulating Rac1 function. Modeling and experiments by others implicate it directly in the regulation of Rac1 activation by nucleotide exchange, and in similar events for Ras [37]–[40]. Our data indicate that Y64 phosphorylation provides a negative input on GTP-binding and cell spreading (Figures 5 and 2, respectively). Interestingly, X-ray crystallography of the Cdc42-RhoGDI complex shows that the Y64 of Cdc42 is in close proximity to lysine residues at positions 43 and 52 of RhoGDI. It is believed that the negative charge induced by tyrosine phosphorylation at Y64 could stabilize the interaction with these two positively charged basic residues on RhoGDI [15], [20]. This hypothesis is supported by our finding that the Rac1-Y64F mutation weakened Rac1 interaction with RhoGDI (Figure 8).

Experimental probing for potential interactions between tyrosine phosphorylation and either constitutive or dominant negative changes in Rac1 activation indicate that there may be separable and combinatorial actions of these two signaling mechanisms with regard to GTP loading, Rac1 targeting to focal adhesions, and cell spreading. The 3-fold increase in GTP loading on Rac1-Y64F as compared with the wild type may be due in part to the fact that the Rac1-Y64F mutant binds more efficiently with Rac1-associated GEFs (Figure 6). Thus, we note an 89% increase in binding with β-PIX, and a more difficult to quantify increase in binding to Tiam1 for Rac1-Y64F as compared with Rac1-WT. Further, the Rac1-Y64D mutant that mimics a constitutively phosphorylated state appears to exert a downward regulatory effect on GTP loading, focal adhesion targeting, and cell spreading efficiency in both constitutively active and dominant negative Rac1 mutants. Taken together with the RhoGDI data, these findings depict a pattern of negative regulation that is a precedented theme in signaling regulation by tyrosine kinases and has been demonstrated in the context of FAK interactions with endophilin A, Src, and MMP-14 [41]. In the case under study here, down-regulation of Rac1 function by FAK and Src may directly oppose and thereby modulate the largely positive effects that these two nonreceptor tyrosine kinases have on lamellipodial extension by other means, e.g. via βPIX phosphorylation and its subsequent increased activation of Rac1.

We have previously demonstrated that Rac1 can localize to focal adhesions and focal complexes at the leading edges of actively evolving membrane ruffles and lamellipodia [21]. This localization appeared to be affected by the activation state of Rac1. MEF expressing the constitutively active Rac1-Q61L mutant showed good focal adhesion targeting accompanied by broad lamellipodia and increased cell size, while cells expressing the dominant negative Rac1-T17N mutant demonstrated the opposite. Data in the present study show that the Y64F mutation did not decrease focal adhesion targeting in HUVEC expressing Rac1-Q61L (Figure 4). In marked contrast, expression of either Y64D or 61L64D was associated with decrements in focal adhesion targeting and lamellipodial deployment, and a limitation of spreading efficiency when compared with cells expressing WT, 61L, or 61L64F Rac1 constructs. Kymography indicated that the changes in lamellipodial formation and cell spreading may result from poor stability of these structures – HUVEC that expressed Rac1-Y64D repeatedly extended lamellipodia, but these structures could not support cell spreading because they were not persistent (Figure 3) (see also [25]). These data are consistent with a role for tyrosine phosphorylation as a negative regulator of the stability of both Rac1 association with matrix adhesion sites and lamellipodial extension. Some of these effects may be mediated by changes in Rac1 interactions with PLCγ which has a known role in lamellipodial extension and persistence [42]. Phe37 and Tyr64 of Rac1 have been shown to bracket Tyr118 of PLC-beta2 to form a series of off-angle, edge-to-face, π-orbital stacking interactions [43]. The negative charge introduced by phosphorylation of Y64 could destabilize this interaction and sabotage lamellipodial development. The PIX- and PAK-binding data presented in Figures 6 and 7 suggest that destabilization of Rac1-PIX-PAK complexes could also contribute to these changes in lamellipodial dynamics (see [21]).

In the context of relatively rapid cycling through phosphorylation and dephosphorylation at the leading edge of a spreading or migrating cell, the negative modulation of adhesion and lamellipodial stability by events at Rac1-Y64 could serve to control cell movement and plasticity. Abrogation of phosphorylation at this site with the substitution of phenylalanine for tyrosine increased the rate of lamellipodial extension (Figure 3). However, the static state of constitutive phosphorylation that is modeled by the Y64D mutation apparently causes severe limitation of stable membrane extension and leads to a contracted phenotype (Figures 2 and 3). This does not, however, immediately trigger apoptosis (Figure S1). Our hypothesis is that the recovery of pY64-Rac1 from cells is complicated by the short time scale of the phosphorylation state, and by the inefficient recovery of Rac1 from cell lysates that is encountered by many workers in this field.

Our investigations of direct interactions between Rac1, Src, and FAK were motivated by our previous insights into FAK augmentation of Rac1 activation during cell spreading, and the Cerione lab's work on the interactions between Src and Cdc42 [20], [21]. The development of purified reagents suitable for the study of interactions between individual kinase-substrate pairs in vitro made it possible to define roles for both Src and FAK in the tyrosine phosphorylation of Rac1 on tyrosine (Figure 9)[22]. Interestingly, these experiments led to identification of Y64 as the major target tyrosine on Rac1 for FAK and Src. This is only one of eight tyrosines in the human Rac1 amino acid sequence, and one of six that are homologous with tyrosine residues in human Cdc42 (together with Y23, Y32, Y40, Y72, and Y154; see Figure 1), yet it is identical to the major Src target identified in Cdc42. In vitro kinase assays showed that when Y64 was mutated to F64 in GST-Rac1, tyrosine phosphorylation was reduced by 92.8% in assays with FAK as compared with the GST-Rac1-WT, and by 62.6% in assays with Src. This discrepancy suggests an interesting difference in the intermolecular interactions between Rac1 and each of these two nonreceptor kinases. The residual tyrosine phosphorylation by Src and FAK that were observed when GST-Rac1-Y64F was used as a phosphorylation substrate suggest the possibility of other tyrosine phosphorylation targets. This possibility was further supported by the presence of tyrosine phosphorylation on EGFP-Rac1-Y64F when it was co-transfected with wild-type Src into SYF cells (not shown). Analysis using the online Phosida database identified Y98 as another possible tyrosine phosphorylation site having specific homology with sites in other substrates for the kinase ALK (anaplastic lymphoma kinase). As in the case of Src-mediated effects on Cdc42, the tyrosine phosphorylation of Rac1 at Y64 changes interactions with RhoGDI (Figure 8)[20]. Our data indicate that abrogation of Y64 phosphorylation on Rac1 via an Y64F point mutation halves Rac1 association with RhoGDI. By inference, phosphorylation at this site may facilitate the binding of membrane-bound Rac1 to RhoGDI. Phosphorylation of Rac1 at Y64 could then have an inhibitory influence on Rac1 activation by interfering with its release from this complex by diacylglycerol kinase **ζ** [44]–[46].

Taken together, the findings reported here are consistent with a possible mode of cross-talk between two cytoskeletal regulating mechanisms that are turned on by integrin engagement during the early phases of cell attachment and spreading on extracellular matrix: the nonreceptor tyrosine kinases Src and FAK, and the Rho family member Rac1. The inhibition of Rac1 by tyrosine phosphorylation in this context appears to represent an addition to the checks and balances that control cell shape and volume during cell spreading.

## Supporting Information

Figure S1 **Expression of Rac1-64D limits cell spreading but does not induce apoptosis.** A. Caspase 3 cleavage was quantified by immunofluorescence as a marker of apoptosis in MEF that were treated for 16 h with 0.5 mM H_2_O_2_. All of these cells were positive (representative cells are shown in the top panels). Scale bar denotes 20 µm. B. In contrast, all of the Rac1-64D-transfected MEF cells were negative for cleaved caspase 3 after 24 h of transgene expression (lower panel). GFP-Rac1-64D is shown in green, anti-cleaved caspase 3 antibody labeling is shown in blue, phalloidin is shown in red, and DAPI is shown in grey scale.
(TIF)

Figure S2 **HUVEC and line scans used to analyze the effects of mutant Rac1 on lamellipodial extension.** Phase images are shown of some of the HUVEC transfected with Rac1-WT, and Rac1-64F, and Rac1-64D that were used for the kymography analysis shown in Figure 3. The line scans used to analyze lamellipodial movements are shown for each cell.
(TIF)
