# Supplementary figures and images for: Tyrosine Phosphorylation of Rac1: A Role in Regulation of Cell Spreading
Source: PLoS One. 2011 Dec 6;6(12):e28587. doi: 10.1371/journal.pone.0028587 (PMC3232246; doi:10.1371/journal.pone.0028587)

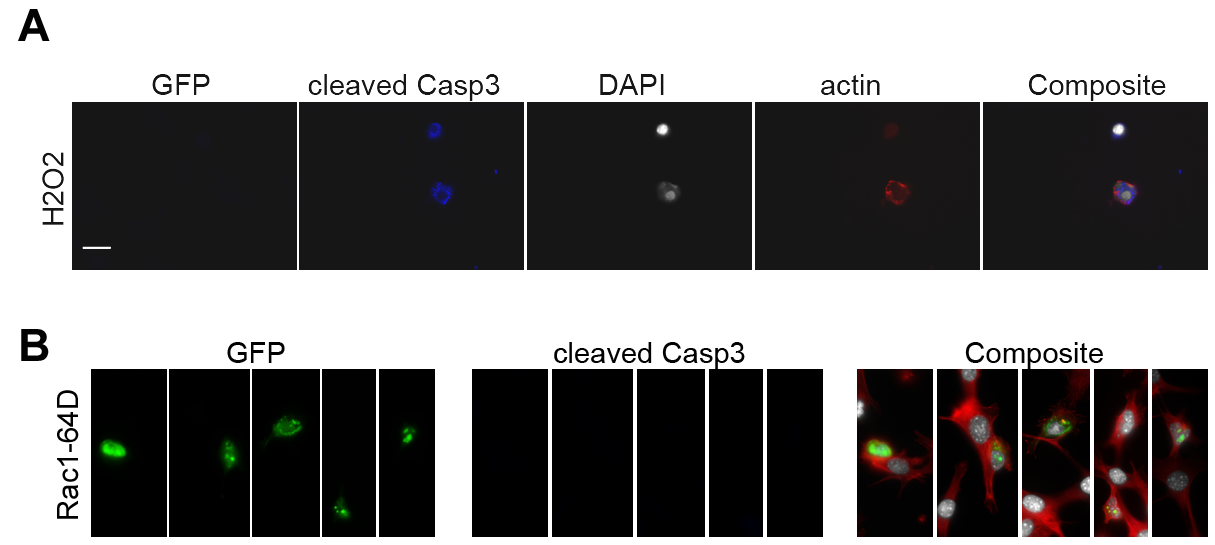

Supplement: Figure S1 — Expression of Rac1-64D limits cell spreading but does not induce apoptosis. A. Caspase 3 cleavage was quantified by immunofluorescence as a marker of apoptosis in MEF that were treated for 16 h with 0.5 mM H2O2. All of these cells were positive (representative cells are shown in the top panels). Scale bar denotes 20 µm. B. In contrast, all of the Rac1-64D-transfected MEF cells were negative for cleaved caspase 3 after 24 h of transgene expression (lower panel). GFP-Rac1-64D is shown in green, anti-cleaved caspase 3 antibody labeling is shown in blue, phalloidin is shown in red, and DAPI is shown in grey scale. (TIF) [file pone.0028587.s001.tif]

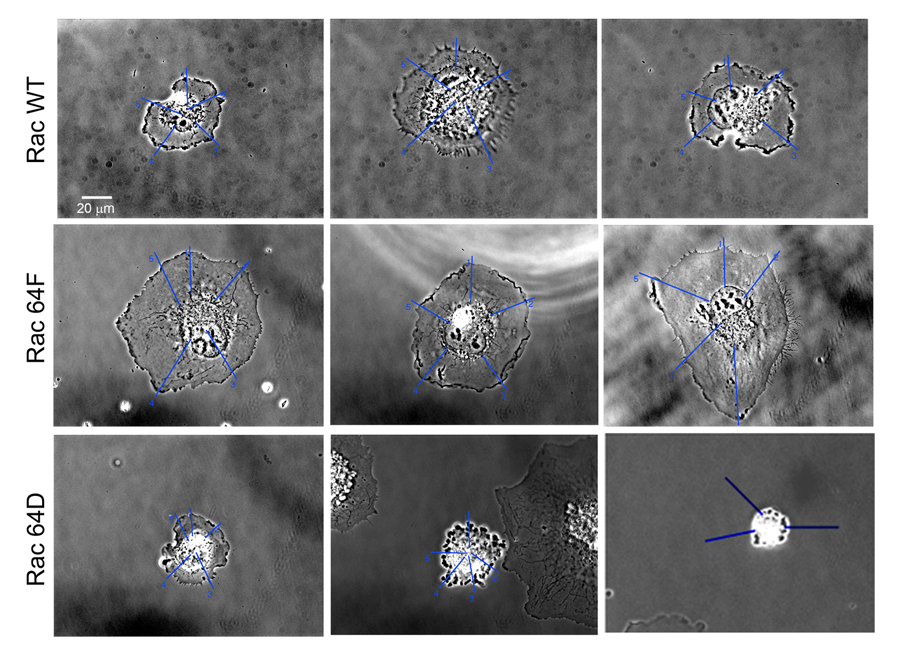

Supplement: Figure S2 — HUVEC and line scans used to analyze the effects of mutant Rac1 on lamellipodial extension. Phase images are shown of some of the HUVEC transfected with Rac1-WT, and Rac1-64F, and Rac1-64D that were used for the kymography analysis shown in Figure 3. The line scans used to analyze lamellipodial movements are shown for each cell. (TIF) [file pone.0028587.s002.tif]
